# Solving the Pervasive Problem of Protocol Non-Compliance in MRI using an Open-Source tool *mrQA*

**DOI:** 10.1007/s12021-024-09668-4

**Published:** 2024-06-11

**Authors:** Harsh Sinha, Pradeep Reddy Raamana

**Affiliations:** 1 Intelligent Systems Program, School of Computing and Information, University of Pittsburgh, Pittsburgh, USA; 2 Department of Biomedical Informatics, University of Pittsburgh, Pittsburgh, USA; 3 Department of Radiology, University of Pittsburgh, Pittsburgh, USA

**Keywords:** Neuroimaging, Quality assurance, Dataset integrity, Protocol compliance, DICOM, Open data

## Abstract

Pooling data across diverse sources acquired by multisite consortia requires compliance with a predefined reference protocol *i.e.*, ensuring different sites and scanners for a given project have used identical or compatible MR physics parameter values. Traditionally, this has been an arduous and manual process due to difficulties in working with the complicated DICOM standard and lack of resources allocated towards protocol compliance. Moreover, issues of protocol compliance is often overlooked for lack of realization that parameter values are routinely improvised/modified locally at various sites. The inconsistencies in acquisition protocols can reduce SNR, statistical power, and in the worst case, may invalidate the results altogether. An open-source tool, mrQA was developed to automatically assess protocol compliance on standard dataset formats such as DICOM and BIDS, and to study the patterns of non-compliance in over 20 open neuroimaging datasets, including the large ABCD study. The results demonstrate that the lack of compliance is rather pervasive. The frequent sources of non-compliance include but are not limited to deviations in Repetition Time, Echo Time, Flip Angle, and Phase Encoding Direction. It was also observed that GE and Philips scanners exhibited higher rates of non-compliance relative to the Siemens scanners in the ABCD dataset. Continuous monitoring for protocol compliance is strongly recommended before any pre/post-processing, ideally right after the acquisition, to avoid the silent propagation of severe/subtle issues. Although, this study focuses on neuroimaging datasets, the proposed tool mrQA can work with any DICOM-based datasets.

## Introduction

Large-scale neuroimaging datasets play an essential role in characterizing brain-behavior relationships. The average sample size of neuroimaging studies has grown tremendously over the past two decades (Bandettini, 2012; Szucs & Ioannidis, 2020). Open datasets like the Alzheimers Disease Neuroimaging Initiative (ADNI) consists of 800 subjects from 50 sites collected over 2-3 years (Petersen et al., 2010), the Human Connectome Project (HCP) (Van Essen et al., 2013) contains 1200 subjects, the Adolescent Brain Cognitive Development (ABCD) study (Casey et al., 2018) includes over 12000 subjects at 21 sites, the Autism Brain Imaging Data Exchange (ABIDE) provides a dataset of 1000 individuals at 16 international sites, and the UK Biobank is following about 100,000 subjects in the UK. These large-scale datasets are acquired over several years, involving multiple sites, with several vendor-specific scanner models.

**Fig. 1:**
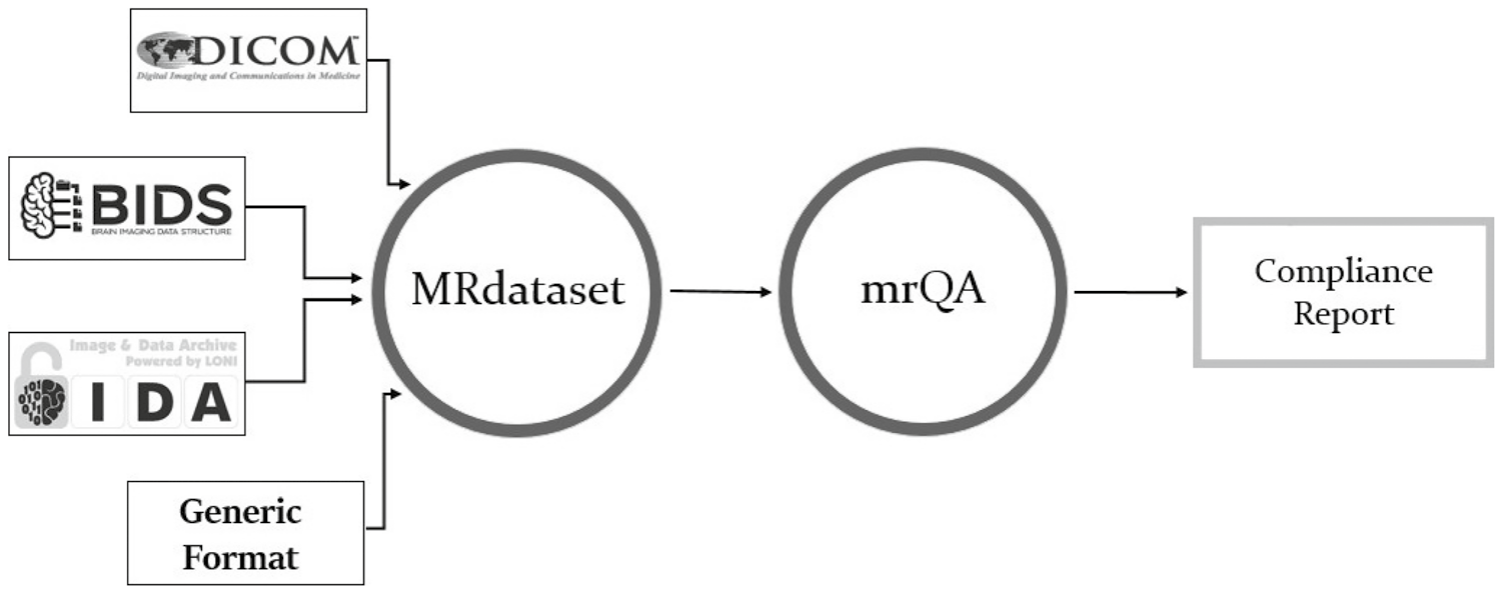
*MRdataset* offers a unified interface to parse & traverse different dataset formats and access acquisition information and metadata e.g. various modalities, subjects, and sessions. This interface is used for generating protocol compliance reports via *mrQA*

A typical MR imaging session consists of multiple modalities (including but not limited to anatomical, functional, and diffusion MRI) along with their corresponding field maps, localizers, and the like for each subject. Imaging data from these modalities provides complementary information about the structural and functional organization. The electronic protocol files generated by scanners (*i.e.*, Exam Card - Philips, Protocol Exchange - GE, or .exar/.edx file - Siemens) include thousands of parameter values for a single session. To use these distinct modalities effectively, it is important to validate the combinations of acquisition protocols *i.e.*, evaluating the reliability of chosen imaging sequences and ensuring that the imaging data is acquired accurately for each subject across all sites and scanners. Neither is it a recommended scientific practice nor is it practical to “hope” for data integrity by manual compliance checks across numerous parameters, given the ever-increasing size of neuroimaging studies, cross-site evaluations, multiple scanners, and varied environments.

As maintenance of imaging protocols in MRI centers is typically an ad-hoc and error-prone process, it often leads to variations in acquisition parameters across different subjects and sessions. For instance, manually uploading protocol configurations on each scanner impacts consistency. Apart from manual adjustments by the MRI technologists on the scanner interface, inconsistencies also emerge due to vendor-specific differences in implementations of imaging sequences, occasional software updates and operational differences across sites that alter the default parameter configuration on the scanner interface. Moreover, technologists often have to make patient-specific changes to the protocol on a session-by-session basis to follow various patient safety and regulatory policies (e.g., maintaining SAR levels below a certain threshold). These adjustments can alter a few other linked parameters owing to the constraints from MRI Physics. It’s the latter changes that are too subtle and often overlooked. Therefore, despite training MRI technologists to ensure protocol compliance, issues of non-compliance can arise and easily go unnoticed due to the fast-paced nature of their job and tight time slots during the imaging session. It is simply impractical to manually verify compliance across multiple sites and scanners, as each MR session has thousands of acquisition parameters.

Even subtle deviations in acquisition parameters can potentially affect the reproducibility of MRI-based brain-behavior studies (Jovicich et al., 2009). Prior works have focused on developing post-processing techniques to reduce the impact of deviations on neuroanatomical estimates (Friedman et al., 2008; Gouttard et al., 2008; Jovicich et al., 2006; Pardoe et al., 2008; Schnack et al., 2004; Fortin et al., 2018). Such post-processing techniques often rely on a large sample size per site to estimate site-specific effects. George et al. (2020) used power analysis to demonstrate that using standardized protocols yields over a two-fold decrease in variability for cortical thickness estimates when compared against non-standardized acquisitions. Therefore, adherence to standardized image acquisition protocols at the scanner is essential for ensuring the quality of MRI-based neuroimaging studies (Jack et al., 2008; Pardoe et al., 2009; Schlett et al., 2016). Otherwise, some subject-specific scans might have to be discarded due to a flawed data collection process, thus reducing the sample size and, consequently, the power of statistical analyses (Button et al., 2013). Yet not much effort has been devoted to eliminating these inconsistencies in image acquisition protocol.

Insufficient monitoring can lead to non-compliance in imaging acquisition parameters, including but not limited to flip angle (FA), repetition time (TR), phase encoding direction (PED), pixel bandwidth (PB), and echo time (TE). When the acquisition parameters are not compliant across scans, it can significantly affect the tissue contrast in T1w/T2w images (Mayerhoefer et al., 2009; Gold et al., 2004). In EPI, co-registration with its structural counterpart becomes difficult if EPI is non-compliant with the field map  (Wang et al., 2017; Jezzard, 2012). In DTI, the images acquired with different polarities of PED cannot be used synonymously as they differ in fractional anisotropy estimates (Kennis et al., 2016). Inconsistencies in image acquisition parameters may implicitly bias the texture in brain images, confounding brain-behavior prediction or phenotypes from brain images (Mayerhoefer et al., 2009). Thus, any analysis conducted without eliminating sources of error in acquisition parameters may reduce statistical power and, in the worst case, may invalidate results altogether, hindering widespread clinical adoption of the experimental results.

**Fig. 2:**
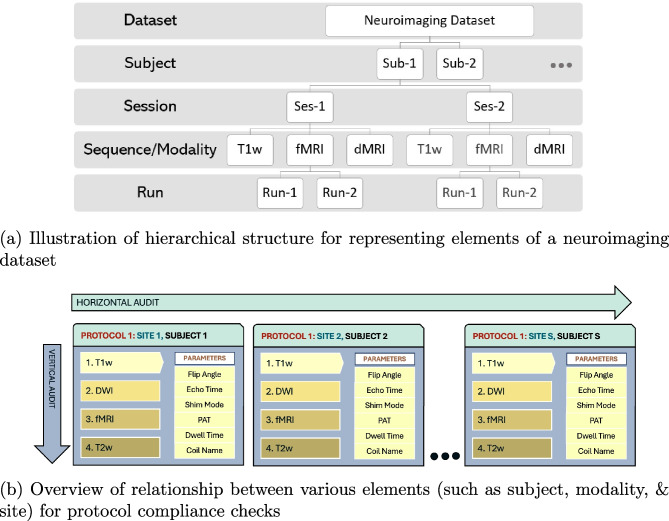
*MRdataset* parses the acquisition parameters for all modalities, subjects, sessions, and runs directly from DICOM headers. Neither does it depend on filename hierarchy nor it expects a particular file organization on disk to accommodate varied configurations in MRI datasets. Then, the parameter values are aggregated to assess protocol compliance for a neuroimaging dataset. We define a horizontal audit to be across all subjects in a given modality (compliant w.r.t a predefined protocol), whereas vertical audit checks if a single subject is compliant across all the acquired modalities

Therefore, we present *mrQA* (and *MRdataset*), a software platform to ensure data integrity in MRI datasets. *mrQA* is designed to aggregate and summarize compliance checks on DICOM images at the MRI scanner itself. Automating the compliance check process, *mrQA* can help reduce the risk of errors and omissions in handling and use of DICOM images. DICOM images have an inherent complex structure, and relying on manual interpretation of DICOM fields is prone to error. For instance, left-right flips are not easy to spot visually. However, the ambiguity can be resolved through an automated software that systematically confirms that the DICOM horizontal flip attribute is same as provided in the reference protocol. The software should seamlessly conduct the verification for each scan, removing the necessity for repetitive manual validation (Glen et al., 2020). Such subtle errors can have serious consequences, especially for brain surgery.

Prior works (Covitz et al., 2022) assessed consistency of acquisition parameters for BIDS datasets. Their work is focused on the execution of BIDS-apps by identifying variations in acquisition parameters. It is important to note that reformatting/validation of BIDS datasets typically occurs years after the data acquisition process has been completed. When non-compliant scans are discovered at a later stage, researchers may have to exclude such subjects/sessions to maintain the reliability of their findings. Therefore, it is important to embrace a mindset of proactive quality assurance *i.e.*, validating the acquired data as soon as possible to minimize data loss and prevent any future non-compliance in acquisition. *mrQA* focuses on continuous monitoring that detects variation in acquisition parameters for DICOM images right away (straight off the scanner) to generate user-friendly reports automatically.

Even though the DICOM format suffers from storage overhead, with complex specifications, DICOM contains complete acquisition metadata with standardized tags. Therefore, it has been the established output format for medical images. In contrast, NIfTI has limited scope for adding important acquisition parameters in the header. The NIfTI format relies on JSON sidecars for storing important acquisition parameters. *mrQA* can discover variations in acquisition parameters in the rawest data format available, *i.e.*, DICOM format. *mrQA* has been developed primarily for DICOM-based datasets, but it also expands its functionality to NIfTI-based BIDS datasets.

An ideal approach is to perform a *near real-time* assessment of protocol compliance, which refers to pre-scanning verification of acquisition parameters for compliance at the scanner itself, so that scans are not acquired with non-compliant parameters to start with. It might be possible that the default acquisition parameters in the scanning interface are inconsistent with the recommended protocol. Such pre-emptive policies can help avoid any non-compliance before provisioning the protocol for initiating the scan. Although, achieving *near real-time* compliance evaluation is our long-term goal, it is a complex endeavor due to the challenges posed by its logistics and the scanner interfaces. Hence, we focus on evaluating compliance after data-acquisition as a first crucial step to provide a critical perspective on the wide diversity of acquisition parameters in open neuroimaging datasets. It is important to note that this exploration is not about finger-pointing for mistakes. Rather, the motivation is to identify common issues of non-compliance and working collaboratively to address them. Towards this end, we assess protocol compliance, or lack thereof, in the The Adolescent Brain Cognitive Development (ABCD) Study dataset (Jernigan, 2017), over 20 datasets on OpenNeuro (Markiewicz et al., 2021) and public DICOM datasets on The Cancer Imaging Archive (TCIA) (Clark et al., 2013).

## Methods

### Overview of *mrQA*

The evaluation of protocol compliance is depicted in two stages as shown in Fig. 1. First, we parse the input dataset to create a data structure that stores the acquisition parameters of all the modalities, subjects, and sessions as shown in Fig. 2 using *MRdataset* (see Appendix A). Then, the acquisition parameters are aggregated and summarized for generating a protocol compliance report (via *mrQA*). An example script for generating compliance reports is provided in Listing 1. Table 3 provides an example of a compliance report generated for a toy dataset.

There can be two types of compliance evaluations - a *horizontal audit* and a *vertical audit*. A *horizontal audit* is focused on assessing parameters for each modality w.r.t. a reference protocol across all subjects in a dataset. A reference protocol is a pre-defined value for each of the acquisition parameters. In a *horizontal audit*, a run is said to be *compliant* if the acquisition parameters for the run are same as the reference protocol. As shown in Fig. 2, a subject may have one or more sessions for each modality (e.g. T1w) and each session has multiple runs. A subject is said to be *compliant* for a given modality if all the sessions for the subject are *compliant* with the reference protocol. Therefore, a subject can be *compliant* for one modality (say T1w), but it might be *non-compliant* for another modality (say T2w). A subject is tagged as *non-compliant* even if a single run is found to be non-compliant. A modality is said to be *compliant* if all the subjects in this modality are *compliant* for all sessions. This means there might be some datasets where none of the subjects are compliant.

A *horizontal audit* is essential to ensure the acquisitions across sessions were performed correctly. However, a *horizontal audit* does not address the interaction between multiple modalities within a given session. In contrast, a *vertical audit* checks for compliance issues across all the modalities for each subject within an imaging session. For example, given a subject, all field maps must be set up with the same field-of-view, number of slices, slice thickness, and angulation as the EPI (Wang et al., 2017). Similarly, shimming method is specific to a subject (Gruetter, 1993). We encourage use of high-order shimming that is consistent across all the subjects in the dataset, especially for spectroscopic experiments (Barker et al., 2010). However, minor deviations in shimming across subjects may not warrant the exclusion of a scan. In addition, vertical audits are helpful in revealing specific scans which are found to be non-compliant across multiple modalities. For instance, a vertical audit can spot navigator slices that might have been erroneously uploaded along with a scan for a subject. We recommend that both *horizontal audit* and *vertical audit* must be enforced to eliminate subtle errors in acquisition protocols.

Further, we advocate a two-pronged approach for checking compliance against a reference protocol. The first is *pre-acquisition* compliance, where the parameters will be checked for compliance against a reference protocol before a scan is performed. And the second step is* post-acquisition *compliance, where the parameters are checked after complete data acquisition, validating the *acquired* dataset for compliance. Ideally, both of these two prongs should be performed to maximize data integrity and to minimize loss *i.e.*, carrying out pre-acquisition compliance checks at initial setup to prevent bad acquisitions in the first place and validating the acquired images with post-acquisition compliance checks to remove any accidental or unknown sources of non-compliance.
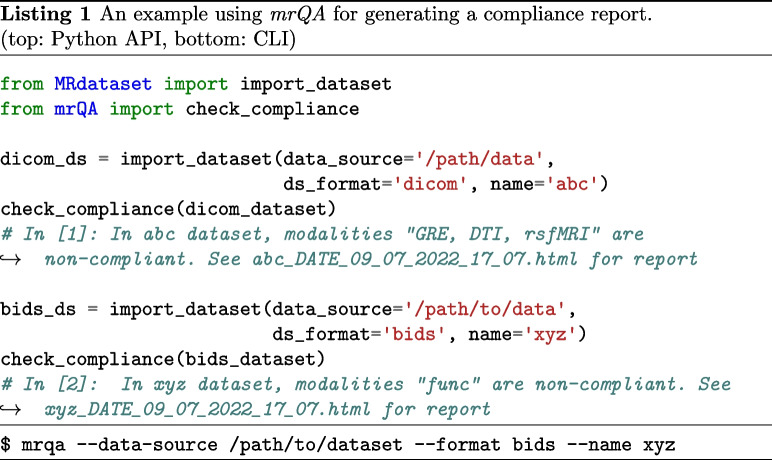


In addition, *mrQA* is also being used for continuous monitoring of DICOM datasets in MR labs. *mrQA* can be set up as a cron job to generate reports at regular (daily/weekly) intervals. Meanwhile, if new sessions are acquired, *mrQA* reads the new DICOM files added since the previous run and generates updated compliance reports for the study. The automatic reporting feature is especially useful to notify researchers about any non-compliance in a timely manner so that corrective action can be taken promptly. An example script is provided in Listing 2.

### Experimental Setup

In this work, we focus on the horizontal audit via post-acquisition compliance to assess neuroimaging datasets for compliance. Assuming that acquisition for most subjects in a given study follows a predefined recommended protocol, *mrQA* infers the most frequent values for each parameter within a modality to construct the *reference protocol*. Then for each subject in the modality, *mrQA* compares the parameter values of each run with the *reference protocol* to determine whether the subject is non-compliant. Finally, each modality is indicated with scores of non-compliance and compliance percentage as shown in Eq. 1.

mrQA is equipped with native support for parsing acquisition protocols in XML files exported from EXAR sources. We recommend using the XML-based “gold-standard” reference protocol exported directly from the scanner in the XML format, if it is available. However, electronic protocol files are not available for public datasets, and therefore we generate compliance reports by inferring the reference protocol, as explained before.

1 $$\begin{aligned} \begin{aligned} \mathrm{non-compliant\; \%}&= \frac{\begin{array}{c}\mathrm{Number\; of\; non-compliant}\\ \mathrm{subjects\; in\; modality}\end{array} \times 100}{\mathrm{Total\; number \;of \;subjects \;in \;modality}} \\ \mathrm{compliant\; \%}&= 100 - \mathrm{non-compliant\; \%} \end{aligned} \end{aligned}$$

By default, *mrQA* checks for absolute equivalence of parameter values. Although, absolute equivalence is preferred to minimize incongruities, minor differences in decimal values may not necessarily be a part of inclusion/exclusion criteria for a subject. Therefore, we analyze the non-compliance percentage by increasing the tolerance level *i.e.*, increasing the acceptable range of variation in parameter values against the reference value as shown in Eq. 2.

2 $$\mathrm{Acceptable\;Range}=\mathrm{R}\pm(\mathrm{t}\times\mathrm{R})$$

where *R* denotes the parameter value in the reference protocol, and *t* denotes the tolerance level. In this work, we adjust the tolerance level *t* between 0.01 to 0.05. Note that changing the tolerance level will not necessarily decrease the non-compliance rate if the deviations are significant, or if parameters are categorical (*e.g.*, PED).
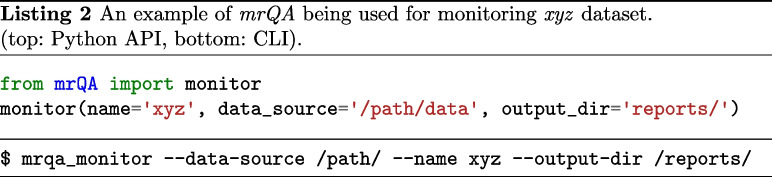

**Fig. 3:**
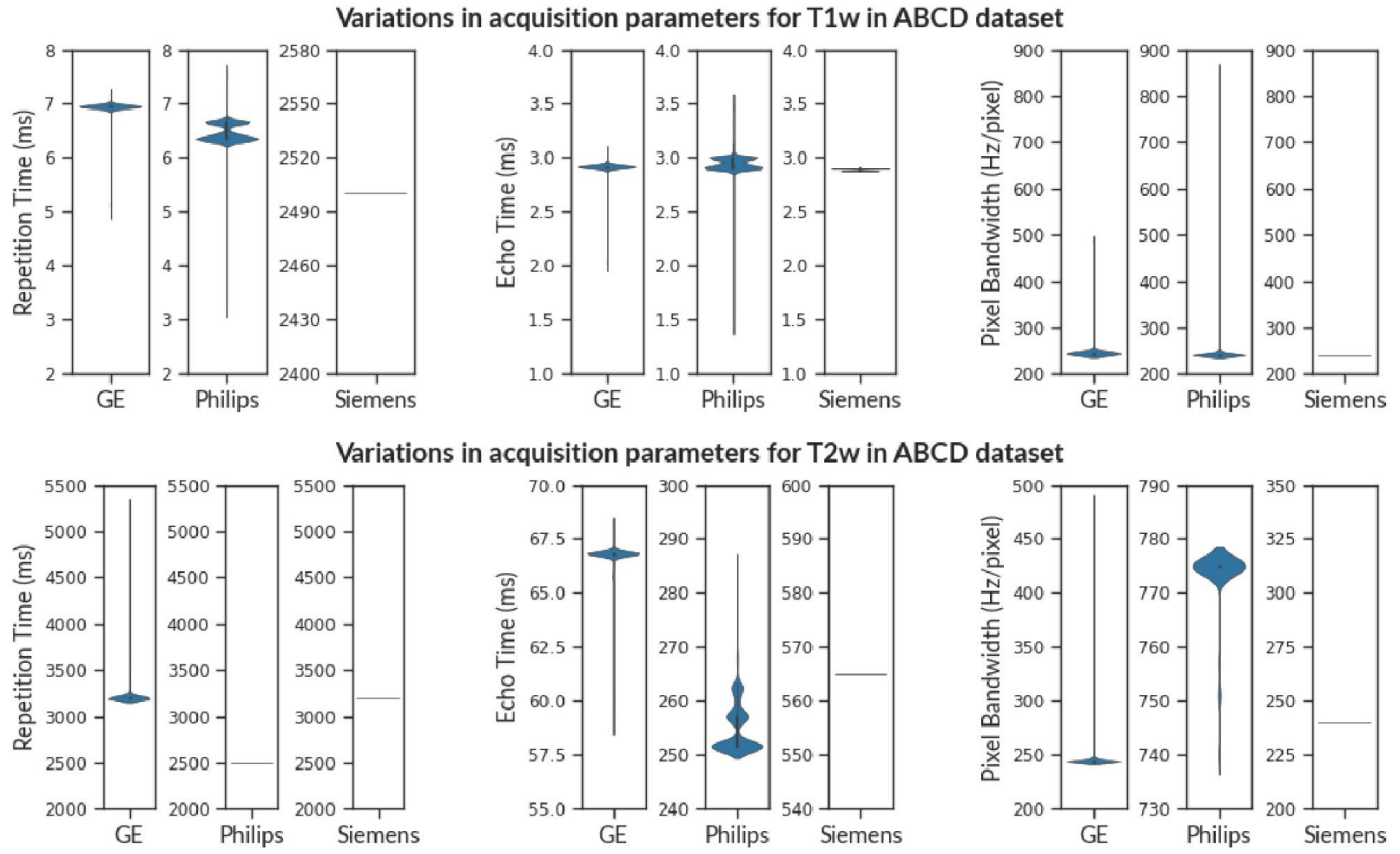
The violin plot shows the variance in Repetition Time (TR), Echo Time (TE), and Pixel Bandwidth (PB) for T1w images (above) and T2w images (below) in the ABCD Dataset. Observe that various vendors have a distinct range of acquisition parameters e.g. Repetition Time (T1w) and Echo Time (T2w). This is because different vendors provide distinct imaging sequences even though the modality might be the same (T1w). Therefore, checking cross-vendor compliance is non-trivial. We observe that scans from Siemens have consistent acquisition parameters in contrast to scans from Philips and GE scanners for both T1w and T2w images

**Fig. 4:**
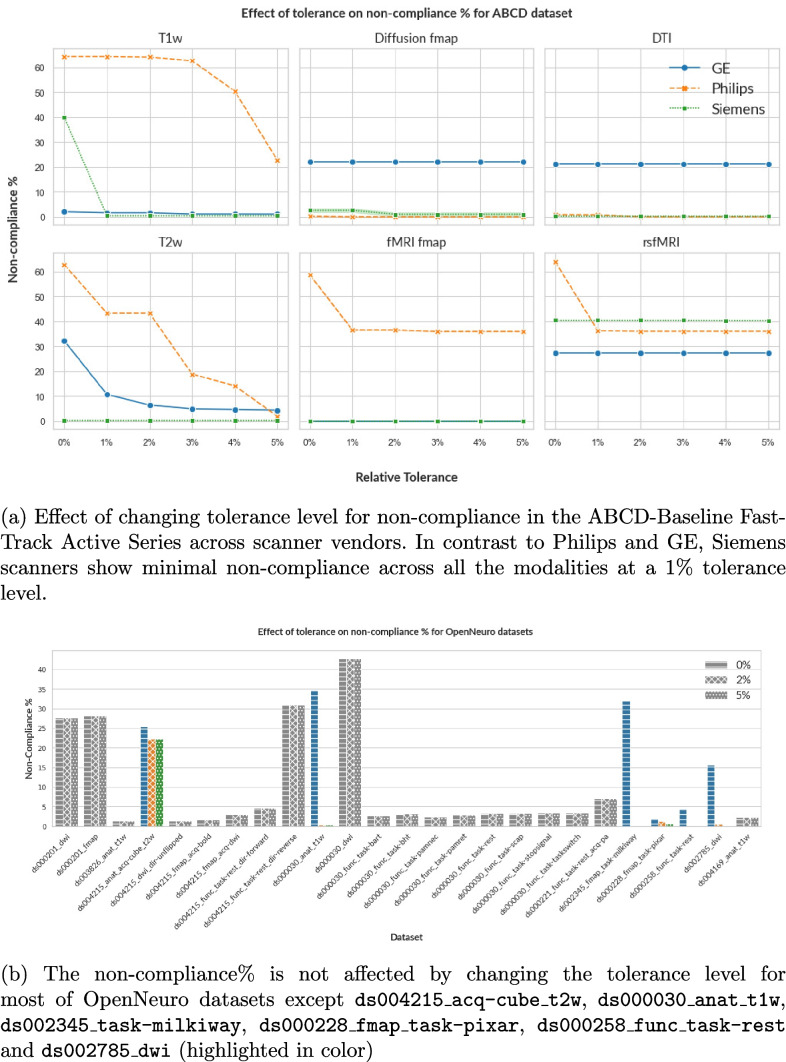
By default, *mrQA* checks for absolute equivalence of parameter values. Given the context of the neuroimaging study, it might be possible to include tolerance in the variation of these acquisition parameters, however, the tolerance level should be best judged by investigators (Sachs et al., 2017). Note that changing the tolerance level will not affect the non-compliance% if the deviation is too large, or if acquisition parameters are categorical

**Table 1:** The table summarizes the compliance report for DICOM images from ABCD-baseline FastTrack Active series. For each of the modality *i.e.*, T1w, T2w, DTI, rsfMRI and field maps (fmap), the table shows the vendor, the percentage of non-compliant & compliant subjects, and the parameters which were found to be non-compliant i.e. Repetition Time (TR), Echo Time (TE), Flip Angle (FA) and Pixel Bandwidth (PB). Some minor cases were observed in Phase Encoding Direction (PED), Phase Encoding Steps (PES), Echo Train Length (ETL), and Shim. In contrast to scans acquired with Philips and GE, images scanned with Siemens exhibit minimal non-compliance across all the modalities. Ensuring compliance in acquisition parameters manually is non-trivial for large-scale multi-site datasets such as ABCD. Automated tools like mrQA can help researchers achieve protocol compliance in a practical manner

**Modality**	**Vendor**	**#Non-compliant Subjects**		**Total Subjects**	**Parameters**	**#Compliant Subjects**	
	GE	59	2.0 %	2941	TE, TR, PB	2882	97.99 %
**T1w**	Philips	980	64.43 %	1521	TE, ETL, PED, PES, PB, TR	541	35.56 %
	Siemens	2883	39.96 %^a^	7214	Shim, TE	4331	60.03 %
	GE	907	32.19 %	2817	TE, TR, PB	1910	67.80%
**T2w**	Philips	916	62.82%	1458	TE, PB	542	37.17%
	Siemens	6	0.08 %	7030	PED, Shim	7024	99.91%
**Diffusion Fmap**	GE	620	22.26%	2785	FA, PB	2165	77.73 %
	Philips	3	0.20 %	1441	PB	1438	99.79 %
**Diffusion Fmap A** $\gg$**P**	Siemens	128	1.81 %	7057	PED, TR, Shim	6929	98.18 %
	Philips	4	0.27 %	1439	PB	1435	99.72 %
**Diffusion Fmap P** $\gg$**A**	Siemens	233	3.3 %	7053	PED, TR, Shim	6820	96.69 %
** fMRI Fmap**	GE	0	00.00 %	2862		2862	100.00 %
	Philips	873	58.7 %	1487	FA, PB	614	41.29 %
** fMRI Fmap A** $\gg$**P**	Siemens	1	0.01 %	7200	Shim	7199	99.98 %
	Philips	875	58.76 %	1489	FA, PB	614	41.23 %
** fMRI Fmap P** $\gg$**A**	Siemens	0	0.00 %	7202		7202	100.0 %
	GE	581	21.32 %	2725	FA, PB	2144	78.67 %
	Philips	11	0.81 %	1343	PB	1332	99.18 %
**DTI**	Siemens	14	0.23 %	5873	PED	5859	99.76 %
	GE	674	27.16 %	2481	PB	1807	72.83 %
	Philips	787	63.98 %	1230	PB	443	36.01 %
**resting-state fMRI**	Siemens	2372	40.34 %	5880	iPAT	3508	59.65 %

^a^There are minor deviations in TE (ms) within the range (2.88, 2.9)

**Table 2:** The table summarizes compliance report for some OpenNeuro datasets that exhibit deviations in acquisition protocol. For each of these datasets, the table shows the modality, the associated suffix for various tasks/acquisition, the percentage of non-compliant & compliant subjects for each modality, and the parameters which were found to be non-compliant i.e. Repetition Time (TR), Echo Time (TE), and Flip Angle (FA). Some minor cases were observed in Phase Encoding Direction (PED), Phase Encoding Steps (PES), Sequence Variant and Pixel Bandwidth (PB). Thus, mrQA provides the ability to automatically discover scanner-related variance in MR datasets. Automatic compliance checks are especially important for large datasets which exhibit non-compliance rate below 1% because manual/ad-hoc checks are ineffective at detecting these subtle issues

**Dataset**	**Modality**	**Differentiating Entities**^b^	**Vendor**	**#Non-Compliant Subjects**		**Total Subjects**	**Parameters**	**#Compliant Subjects**	
	dwi			21	27.63%	76	PB	55	72.36%
**ds000201**	fmap		GE	24	28.23%	85	PED, PB, TR	61	71.76%
**ds003826**	anat	t1w	Siemens	2	1.47%	136	PES, SV	134	98.52%
	anat	acq-cube_t2w		39	25.49%	153	TE, TR	114	74.5%
	dwi	dir-unflipped		2	1.39%	143	PB	141	98.6%
	fmap	acq-bold		1	1.69%	59	PED	58	98.3%
	fmap	acq-dwi		2	3.03%	66	PED, PB	64	96.96%
	func	task-rest_dir-forward		6	4.61%	130	FA, PED	124	95.38%
	func	task-rest_dir-reverse		40	31%	129	FA, PED	89	68.99%
**ds004215**	perf	asl	GE	1	0.7%	142	TR	141	99.29%
	anat	t1w		92	34.71%^a^	265	PES, PB	173	65.28%
	dwi			112	42.74%	262	PB, TR	150	57.25%
	func	task-bart		7	2.66%	263	PED	256	97.33%
	func	task-bht		8	3.1%	258	PED	250	96.89%
	func	task-pamnec		5	2.41%	207	PED	202	97.58%
	func	task-pamret		6	2.88%	208	PED	202	97.11%
	func	task-rest		9	3.35%	268	PED	259	96.64%
	func	task-scap		9	3.35%	268	PED	259	96.64%
	func	task-stopsignal		9	3.38%	266	PED	257	96.61%
**ds000030**	func	task-taskswitch	Siemens	9	3.38%	266	PED	257	96.61%
	fmap	acq-GE		1	0.31%	317	PED	316	99.68%
	fmap	acq-SE		1	0.44%	227	PED	226	99.55%
**ds000221**	func	task-rest_acq-PA	Siemens	14	7.07%	198	TE	184	92.92 %
**ds002345**	func	task-milkway	Siemens	17	32.07%^a^	53	TR	36	67.92 %
**ds000228**	func	task-pixar	Siemens	3	1.93%	155	FA	152	98.06 %
**ds000258**	func	task-rest	Siemens	4	4.49%^a^	85	TR	85	95.5%
**ds002785**	dwi		Philips	33	15.63%^a^	211	TR	178	84.36%
**ds004169**	anat	t1w		27	2.24%	1202	FA, PES, PB, TR	1175	97.75%
	func	task-nback		3	0.25%	1189	FA, PED	1186	99.74%
	func	task-rest	Siemens	2	0.19%	1029	FA, PED	1027	99.8%

^a^There are minor differences in parameter values. See dicussion

^b^For BIDS datasets, entities correspond to an altered acquisition parameter

We focused on evaluating public datasets as they often serve as a benchmark for neuroimaging analyses. Using *mrQA*, we evaluated three distinct collections of neuroimaging datasets for protocol compliance. First, we evaluated DICOM images from the ABCD Dataset (Jernigan, 2017) as it provides a unique opportunity to test on a large and diverse sample of over 11,000 subjects (Table 1). Secondly, we utilized 20 large BIDS datasets publicly available on OpenNeuro (Table 2). The datasets were chosen based on their size and availability of JSON sidecar files. Finally, we analyzed DICOM datasets available on The Cancer Imaging Archive (TCIA) (see Appendix C).

We analyzed ABCD-baseline scans for 4 modalities, namely T1w, T2w, DTI, resting-state fMRI, and associated field maps (referred to as fmap), as shown in Table 1. We analyze DICOM images from the ABCD FastTrack Active Series as it closely represents the unprocessed dataset with the most-complete information (closest to the scanners). We assume that all the data collected so far has been acquired with a single protocol as published in Table 2 in  Casey et al. (2018) but we are aware that this protocol might have changed slightly over the years for various reasons. As these details are currently not accessible to us during our analysis of the dataset as a whole, we analyzed it as it was shared. If we redo the analyses accounting for such approved intentional changes in the reference protocol, our results are likely to change and we may see different levels of non-compliance. To accommodate such intentional changes, it is best to run *mrQA* on subsets with a single fixed reference protocol for an accurate estimation of non-compliance in the dataset.

OpenNeuro (Markiewicz et al., 2021) is a data archive dedicated to open neuroscience data sharing based on FAIR principles (Wilkinson et al., 2016). Table 2 presents some of the datasets which exhibit non-compliance in acquisition parameters. Due to the absence of standard acquisition metadata in NIfTI files, we rely on associated JSON sidecar files for evaluating protocol compliance on NIfTI-based datasets.

## Results

### Evaluation of ABCD dataset

We observed that T1w MRI scans from Philips scanners exhibit non-compliance of 64.43%. As shown in Fig. 3, the echo-time (TE) varies in the range (1.4 ms, 3.56 ms) for Philips scanners, even though structural scans are not multi-echo in general. Similarly, T1w images from the GE scanner have minor issues of non-compliance in TE, TR, and PB. T1w scans from Siemens scanners exhibit some minor issues in TE and shim. Although echo time varies for 39.96% of the subjects, there are only minor deviations within the range (2.88 ms, 2.9 ms).

Similar to T1w images, we observe that 62.82% of subjects are non-compliant for T2w scans from Philips scanners. Figure 3 shows that TE varies in the range (251.49 ms, 285.23 ms) while PB (Hz/pixel) varies between (740, 775). We observe considerable non-compliance (32.19%) in TE, and TR values from GE scanners for T2w images. TE varies in the range (59.1 ms, 68.2 ms) while TR varies in the range (3200 ms, 5297 ms). In contrast, Siemens scanners exhibit minor issues in PED and shim for only 0.08% of subjects.

Table 1 shows the assessment of field maps (fmap) in the ABCD dataset. The subjects are stratified into vendor and PED-specific groups as per information in the DICOM header. Often neuroimaging experiments consist of both A$\gg$P and P$\gg$A scans to reduce susceptibility artifacts (Irfanoglu et al., 2012). Therefore, the scans will not have a unique PED across all scans. To avoid misinterpretation, compliance checks should be performed within these sub-groups of A$\gg$P and P$\gg$A scans. Note that in the ABCD dataset, Siemens and Philips scanners had distinct field maps each annotated with a PED (AP/PA). However, such annotation was absent in field maps from GE scanners. The field maps intended for Diffusion Images should not be compared to the field maps intended for fMRI images. This information is not automatically captured in DICOM images and should be annotated manually after acquisition.

We observe that both the field maps and Diffusion images from GE scanners have two distinct values of flip angles i.e. 77^∘^ and 90^∘^. Even though fMRI field maps from Philips are acquired with flip angle values of 52^∘^ and 90^∘^, the resting-state fMRI scans were acquired only with a flip angle of 52^∘^. The report indicates that these subjects don’t comply with a single predefined value for flip angle. We choose to flag this issue, however, whether it is an issue or a study requirement would be best judged by the investigators of the study(Provins et al., 2023; Reynolds et al., 2023). In contrast, field maps acquired with Siemens scanner have a flip angle of 90^∘^, and some minor issues in Shim, PED, and TR. We also observed that the Table 2 from Casey et al. suggests that parallel imaging was turned off for fMRI sequences acquired with Siemens scanners. But our results show that 60% of subjects were acquired using SENSE.

Figure 4 shows how increasing the relative tolerance level affects the level of non-compliance for T1w, T2w images, and field maps in the ABCD dataset. For T1w and T2w images, the percentage of non-compliance drops close to zero (except for T1w Philips), indicating that the variations lie within 5% tolerance. For T1w from Philips scanners, TE and TR varies beyond the 5% tolerance range of (2.85, 3.15) w.r.t. reference value of 3 ms and (6.33, 6.99) w.r.t. reference value of 6.66 ms, respectively. This results in the non-compliance rate of 22.68% at 5% tolerance level. As the tolerance level is raised from 1% to 5%, the non-compliance rates for diffusion field maps (GE) and fMRI field maps (Philips) show no further decline beyond 22.26% and 35.91%, respectively. This observation can be attributed to large deviations in parameters (such as flip angle and pixel bandwidth) from their reference value, exceeding the 5% tolerance limit.

### Evaluation of OpenNeuro datasets

Table 2 shows evaluation of protocol compliance for OpenNeuro datasets after stratifying modalities by entities such as *task* and *acquisition*. Note that compliance checks should be performed after stratification into coherent clusters as same sequences are often acquired multiple times with varying acquisition parameters for each subject e.g. DTI scans with different PED (A$\gg$P, P$\gg$A) or separate cognitive/behavioral tasks captured with different acquisition protocols in an fMRI study.

We observed that a lot of subjects in datasets such as ds002843, ds000117, ds000228, ds001242, ds004116, ds003647, and ds002345 were missing crucial parameters (such as PED, magnetic field strength, echo train length) from their respective JSON sidecar. We observe that each of the OpenNeuro datasets export a varying set of acquisition parameters because, unlike DICOM tags, JSON sidecar is not standardized. If there is a considerable level of non-compliance, the dataset can be explicitly standardized before it is used for analyses. However, the standardization would have limited validity due to missing acquisition parameters which might impact the reliability of results. Therefore, we recommend that compliance should be checked using DICOM images, which contain complete acquisition metadata with standardized tags.

We also observed that often the same subjects are tagged as non-compliant across several parameters. This can help in identifying consistent patterns in sources of non-compliance. For example, if the same subject is found to be non-compliant for TR and flip angle in T2w FLAIR sequences, this may indicate that the subject was not comfortable inside the scanner, and therefore SAR was adjusted by reducing flip angle and increasing TR value (Allison & Yanasak, 2015). Therefore, adequate support may be provided to the particular subject during any further scans to ensure compliance. We found this pattern in several datasets such as ds003826, ds004169, ds000221, ds000030, ds000201, ds004215, and ds000258. Such patterns might also be helpful in identifying particular sites, or scanners that might be the cause of non-compliance.

**Fig. 5:**
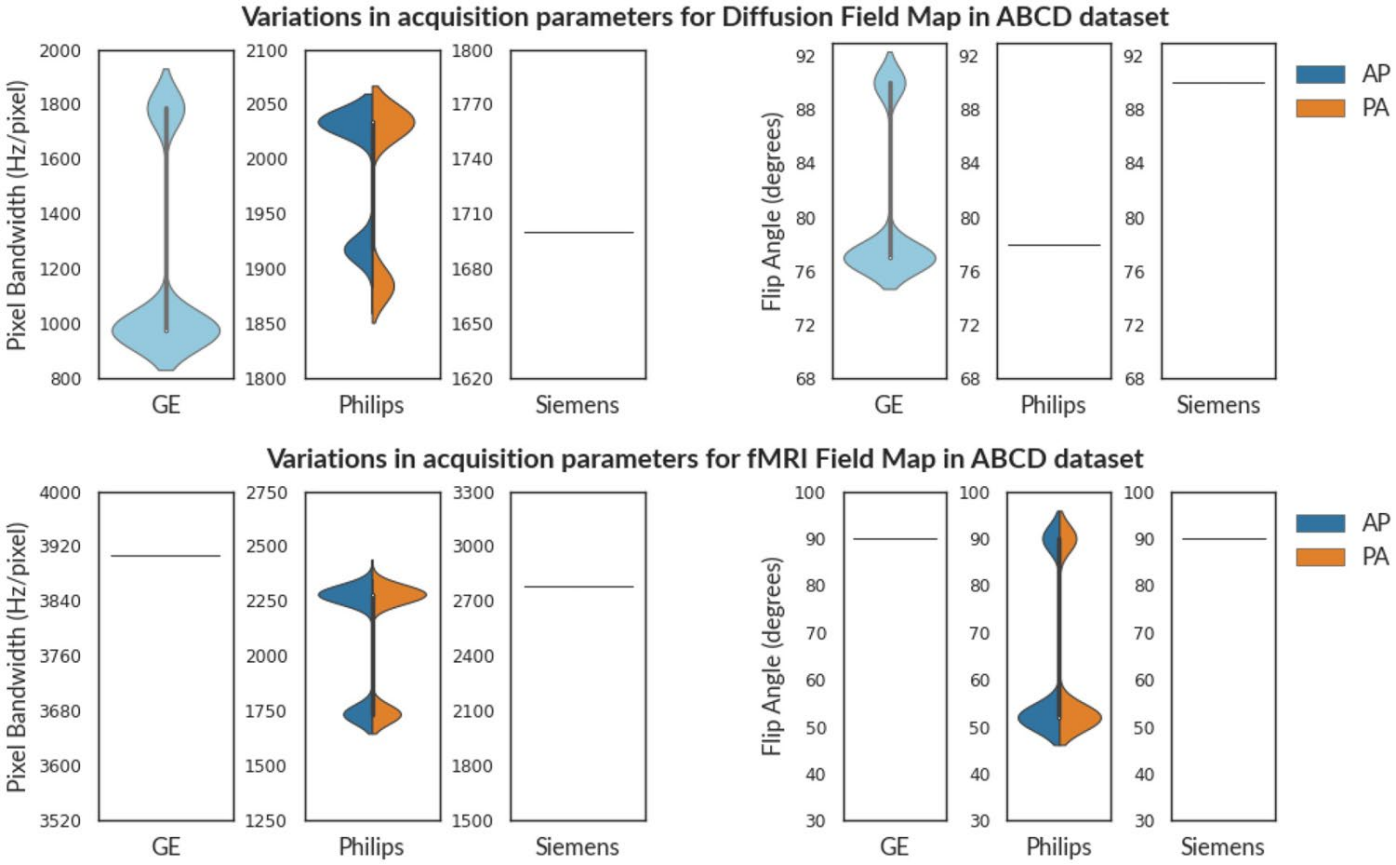
The violin plot shows the variance in Flip Angle(FA) and Pixel Bandwidth (PB) for Diffusion (above) and fMRI (below) field maps in the ABCD Dataset. Siemens and Philips scanners had distinct fieldmaps each annotated with a PED (**AP**/**PA**). However, sequences from GE scanners (denoted by **cyan**) were not annotated in the ABCD dataset. In contrast to scans from Philips and GE scanners, MR scans from Siemens have consistent acquisition parameters across both diffusion and fMRI field maps

**Fig. 6:**
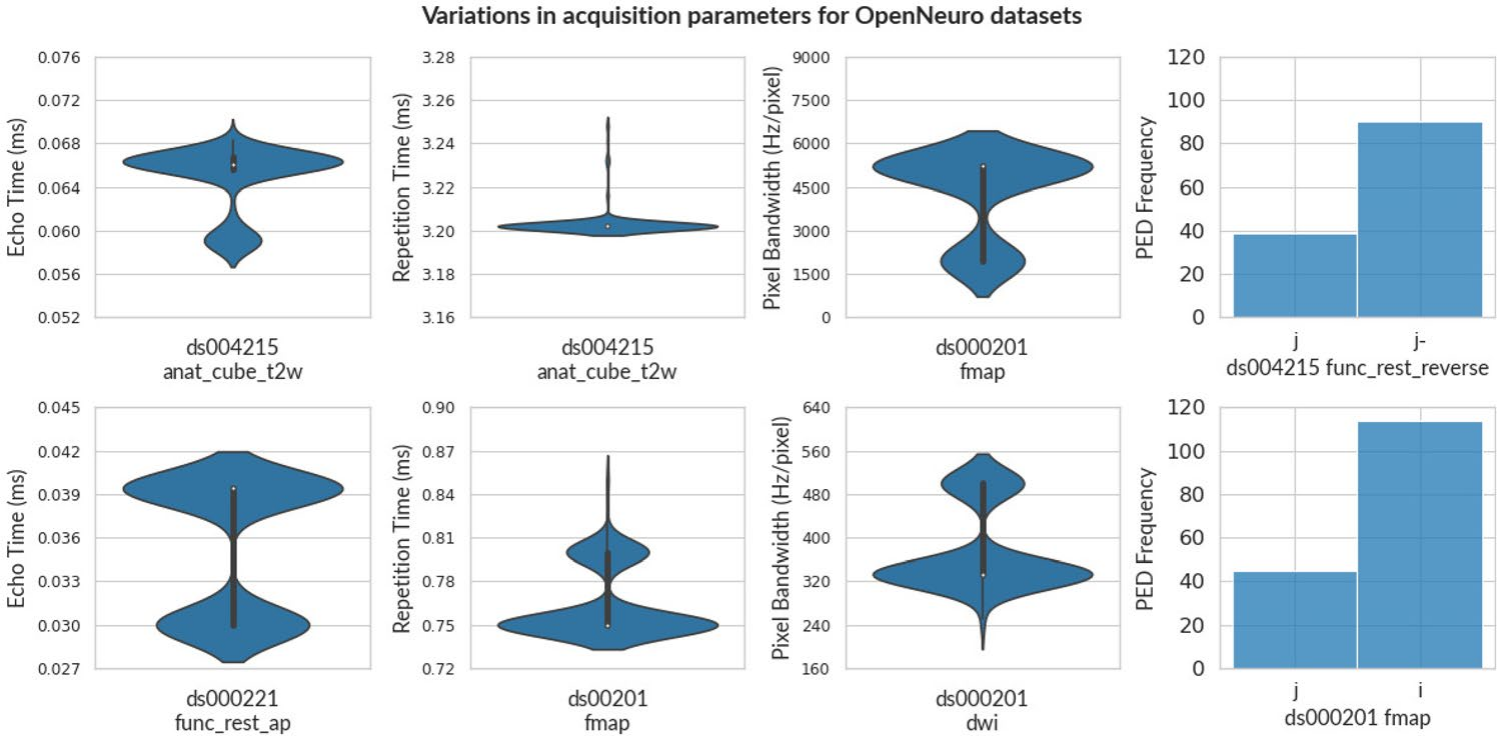
The violin plot shows the variance in Echo Time, Repetition Time, and Pixel Bandwidth for some OpenNeuro Datasets. For violin plots, the width represents the frequency at different levels of each parameter. A histogram chart shows the number of scans for each PED. Note that, even though the entity label specifies PED as *reverse* for ds004215, PED is not consistent. This indicates that ensuring compliance is an arduous process, and issues of non-compliance can be overlooked even after careful effort in data acquisition

Figure 6 provides a visual representation of variance in some of the important acquisition parameters such as TE, TR, PB, and PED across a few OpenNeuro datasets. Further, we evaluate the non-compliance of each of these datasets after increasing the tolerance level as shown in Fig. 4. We observe that the percentage of non-compliance decreases for 6 datasets only (shown in color), while the percentage of non-compliance for all other datasets is not affected (shown in gray) due to large deviations from the reference beyond the 5% tolerance level or if the non-compliant parameters are categorical (e.g. PED).

## Discussion

We briefly discuss how deviations in acquisition parameters affect images (see Appendix D for further information). For instance, the flip angle affects the RF signal of the cycle, thereby affecting the signal intensity (Sandmann et al., 2016; Balezeau et al., 2011; Lutterbey et al., 2007; Gonzalez-Castillo et al., 2011). Figure 5 shows variation in flip angle in field maps for the ABCD dataset. Similarly, timing parameters (such as TE, and TR) influence tissue specific response in anatomical images and BOLD response in functional images (Chen & Glover, 2015; Feinberg and Setsompop, 2013; Poldrack et al., 2008). Figures 3 and 6 show the variance of TE and TR for the ABCD dataset and OpenNeuro datasets, respectively. We also observed that datasets (such as ds004116 and ds004114), aggregated images from various field strengths, (4.7 T - 17.15 T) and (3 T - 14.1 T), respectively. In context to texture features, images with varying field strength cannot be used interchangeably (Ammari et al., 2021). A study may require multiple scans with varying PED to eliminate susceptibility artifacts (Jones & Cercignani, 2010; Le Bihan et al., 2006; Irfanoglu et al., 2012), however it also leads to significant differences in fractional anisotropy estimates (Kennis et al., 2016; Tudela et al., 2017). Figure 6 shows a histogram to visualize the apportionment of PED for ds004215 and ds000201 datasets.

Prior works have measured the effect of various acquisition protocols on texture analysis (Carré et al., 2020; Chirra et al., 2019; Bologna et al., 2019), to evaluate which features are stable against changes in acquisition protocols. Some parameters such as TR and TE do not affect the shape and size of the image, but they affect uniformity in grayscale intensity (Mayerhoefer et al., 2009). It is evident that different acquisition protocols can affect data distribution, reducing the reliability of the extracted features and consequently increasing the bias of downstream statistical analyses (Schurink et al., 2022). Thus, special attention must be attributed to harmonization across scanners, and acquisition protocols (Mali et al., 2021) before any feature extraction (Li et al., 2021). Harmonization is possible only if we know that the data exhibits variation in imaging acquisition protocol. In cases when sources of non-compliance are unknown, data cannot be categorized into clusters, and it would be difficult to perform data harmonization. Thus, *mrQA* can play a pivotal role in establishing data integrity by discovering sources of non-compliance allowing the investigators to perform harmonization, if required.

As we progress towards algorithms that are able to learn features automatically (e.g. deep learning), it is even more important to ensure that the derived image features are stable with respect to variations in acquisition parameters (Mayerhoefer et al., 2009). Without acknowledging these sources of variation in acquisition parameters, the statistical results might be subject to confounding which can obscure or exaggerate the effects of interest (Geirhos et al., 2020), leading to misinterpretation of statistical results.

Further, we explore the issue of non-conformance in parameters w.r.t. vendors. As compared to Philips and GE scanners, MRI scans acquired with Siemens scanners in the ABCD dataset are observed to be consistent achieving more than 99% compliance over 7000 subjects both in T2w and field maps. We observed that MR scans from Siemens scanners were performed only on Prisma scanners with the same software version (*syngo MR E11*). In contrast, scans for Philips were performed on Achieva dStream and Ingenia models, and the GE scans were executed on MR750 and DV25-26 (Casey et al., 2018). Furthermore, both GE and Philips scans had differences in software versions. The difference in hardware and soherftware versions might have been a potential cause of non-compliance in acquisition parameters for Philips and GE scanners. Furthermore, the differences in compliance across vendors can also be consequence of variability in level of maintainence, quality control and operational differences across sites. However, our findings are consistent with those reported by the ABCD-BIDS Community Collection (ABCC)  (Feczko et al., 2021). They observed a relatively high post-processing quality control failure rate, particularly for images derived from GE and Philips scanners.

Although MRI scanners from different vendors function on the same underlying principles, image sequences can have significant differences in gradient strengths, RF pulse sequences, and timing parameters (Okada et al., 2011). In addition, each vendor uses different software and methods to reconstruct the images from *k*-space. Therefore, these sequences are denoted with specific names and abbreviations. For example, Siemens scanners provide an SPACE imaging sequence, while Philips scanners provides VISTA imaging sequence  (Mugler, 2011). Both these are 3D TSE sequences and can create T1w images, however significant differences in hardware and software make it non-trivial to compare scans across vendors due to vendor-specific differences. It is better to stratify scans w.r.t. a vendor to avoid any misinterpretations. This problem becomes particularly relevant for multi-site studies where scans are acquired using multiple scanners with potentially differing acquisition protocols. Therefore, the subjects are stratified into different vendor-specific sub-groups in Table 1 as per the information in the DICOM header.

We observed that scanners from various vendors (*e.g.*, Siemens, GE, Philips) differ in terms of units of measurement and numerical range even for the same parameter. Furthermore, the definition of certain parameters may also vary across vendors based on the particular imaging sequence used. For instance, Field-of-View (FoV) is typically measured in millimeters in Siemens/Philips scanners however GE use centimeters. While analyzing the ABCD dataset, we observed that the TR for Siemens scans was in the range of 2000-4000 ms, but for Philips scans the range was 6-7.5 ms as shown in Fig. 3. The precise details of these differences are stored in Exam Card (Philips), Protocol Exchange (GE), or .exar/.edx file (Siemens) generated by corresponding software. Even though much of the information is available in DICOM metadata, inclusion of these electronic protocol files would allow all scanners to have a uniform acquisition protocol loaded into their system without manual intervention, thus eliminating any potential sources of error across various sites and scanners (Szczykutowicz & Siegelman, 2015). mrQA is equipped with native support for parsing acquisition protocols in XML files exported from EXAR sources. However, automatic cross-vendor compliance is very difficult due to the lack of standardized open-source tools that can effectively read/write and convert proprietary formats from different vendors.

Finally, we discuss current limitations and future directions for the development of *mrQA*. *mrQA* extracts certain acquisition parameters such as the shimming method, PAT, and multi-slice mode from Siemens private headers. mrQA skips the private header while reading DICOM images from GE and Philips scanners. Therefore, *mrQA* at its current stage cannot discover non-compliance in parameters present in private headers for GE/Philips scanners.

Note that the DICOM header doesn’t contain any information beyond the specifications of the MR scanner, for example - variations in duration/intensity of visual stimuli used for measurement of neural responses, reactivity measurements such as CO2 inhalation or acetazolamide infusion (Clement et al., 2022), hardware configurations, head motion and distortion artifacts (Esteban et al., 2017). Therefore, checking compliance in the DICOM header may not be sufficient to achieve QA, instead it is equally important to flag and deal with such issues before deeming an MR session/subject “valid” for inclusion in the neuroimaging study (Taylor et al., 2023).

As of now *mrQA* checks for compliance in a subset of acquisition parameters (in DICOM header) as shown in Table 3. However, there can be other potential sources of non-compliance (Inglis, 2015; Poldrack et al., 2008), for instance, field of view or temporal resolution. However, these parameters don’t have a standard DICOM tag across all scanner vendors. Therefore, it is not always possible to extract these parameters from proprietary vendor-specific private headers to check for compliance. While it’s true that our analysis is primarily centerd around the more obvious acquisition parameters, that are selected after careful consultation with relevant stakeholders such as the MR physicists, technologists and investigators. However, this is not necessarily a limitation of our study. The primary objective of this exploration is to demonstrate the capabilities of automated protocol compliance using *mrQA*(and *MRdataset*). *mrQA* is fully extensible and as additional parameters are integrated (which can easily be added by users as they deem necessary), it is indeed likely that the percentage of non-compliance may increase. Nonetheless, the current analysis represents a crucial first step in raising awareness of the prevalence of non-compliance in MR research. By highlighting existing issues and demonstrating the utility of automated compliance assessment tools, we aim to emphasize the imperative need for improved standardization and reporting practices in the field of MR research.

## Conclusions

A critical aspect of MR imaging is adherence to the recommended protocol which would enhance the validity and consistency of acquired images. However, we demonstrate the pervasive problem of protocol non-compliance based on analyses of many open datasets from OpenNeuro and the ABCD dataset. Secondly, inconsistencies should be checked promptly so that corrective measures can be taken to minimize differences in acquisition parameters over the entire project timeline. It is non-trivial to maintain protocol compliance in imaging acquisition parameters, especially for large-scale multi-site studies. Monitoring compliance would make us much more familiar with our own data, enabling us to draw meaningful conclusions while considering potential biases, confounds, or anomalies that impact the quality of statistical analysis.

Therefore, we propose an open-source tool, *mrQA* (and *MRdataset*) which can summarize and aggregate acquisition parameters to discover any issues of protocol non-compliance. Apart from generating compliance reports, mrQA can be set up for continuous monitoring of acquired DICOM images on a daily/weekly basis. We believe that it is important to embrace a mindset of proactive quality assurance to weed out any source of inconsistencies at the scanning interface itself rather than waiting for the end-of-analyses to catch confounding. Adopting such an approach before organizing files in a suitable directory structure (e.g. BIDS) will save time and effort.

The long-term goal is to analyze DICOM images in *near real-time* to identify and fix any issues of non-compliance at the scanner itself. As we move towards even larger datasets, automated imaging QA would be critical for dataset integrity and valid statistical analyses. *mrQA *can help automate this process, as we move towards practical, efficient, and potentially real-time monitoring of protocol compliance.

## Information Sharing Statement

Data sharing is not applicable to this article as no new data were created in this study. Data used in the preparation of this article are publicly available at www.nda.nih.gov (ABCD), www.openneuro.org (OpenNeuro) and www.cancerimagingarchive.net (TCIA). The software package is available on the Python Package Manager (PyPI) at https://pypi.org/project/mrQA and its source code is publicly available at https://github.com/Open-Minds-Lab/MRdataset and https://github.com/Open-Minds-Lab/mrQA. The software documentation is hosted at https://open-minds-lab.github.io/MRdataset/ and https://open-minds-lab.github.io/mrQA/.

## Data Availability

No new data were created in this study. Data used in the preparation of this article are publicly available at www.nda.nih.gov (ABCD), www.openneuro.org (OpenNeuro) and www.cancerimagingarchive.net (TCIA).

## Appendix A: Creating a Unified Interface *(MRdataset)*

Experimental neuroimaging data is hugely diverse and may be structured differently according to study design or clinical protocol, especially the stimulus, behavioral response, and interventions. Thus, any tool must address the fundamental capability of representing and manipulating common dataset formats. *MRdataset* provides a unified interface to simplify the traversal of neuroimaging datasets by adopting modular classes for each dataset format as well as different levels in the hierarchy, such as modalities, subjects, sessions, and runs (Lindquist & Wager, 2015) as shown in Fig. 1. *MRdataset* infers information about the hierarchical structure directly from the DICOM headers. *MRdataset* doesn’t rely on filenames or expect a particular idiosyncratic organization of files to process various configurations. It provides a simple modular interface to improve the use and accessibility of neuroimaging datasets.

MRdataset provides a consistent set of methods for data access irrespective of the dataset format (such as BIDS, and DICOM). In the future, we expect to extend the *MRdataset* dataset class to support other data formats, such as LONI IDA, but these were not included in the initial design. In addition to the desired unified interface, *MRdataset* performs basic validation to reject localizers, head scouts, and phantoms. Keeping these considerations in mind, the package is written in Python, and it uses *pydicom* (Mason et al., 2022) for reading DICOM images. Python is becoming the de facto standard for scientific applications as it provides community support and easy extensibility for the future (Raamana, 2018).

## Appendix B: Example of a Compliance Report

An HTML report is generated for a toy dataset that presents complete information in a concise manner (as shown in Table 3). The report has two parts - a summary view which gives a brief assessment of protocol compliance for all the modalities. Following the summary, each modality is accompanied by a detailed view. There are two non-compliant subjects, *sub-566* and *sub-879*, for the modalities GRE and DTI, respectively. Subject *sub-566* has non-compliant pixel bandwidth, while subject *sub-879* has an non-compliant PED. For instance, pixel bandwidth in the reference protocol is 350, but subject *sub-566* has a pixel bandwidth of 485 in sessions 6, 7, 8, 9, and 10. The complete compliance report contains reference protocols for each modality and corresponding details about sources of error in acquisition parameters.

## Appendix C: Evaluation of DICOM Datasets on TCIA

Although we focus primarily on neuroimaging datasets, *mrQA* can analyze DICOM-based datasets for other organs also. We also analyze three public DICOM datasets available on The Cancer Imaging Archive (TCIA) (Clark et al., 2013), namely Rembrandt (Scarpace et al., 2019), TCGA-GBM (Scarpace et al., 2016) and TCGA-LGG (Pedano et al., 2016). For Rembrandt, we observe issues of non-compliance in TR. Many scans had missing values for crucial parameters like magnetic field strength, pixel bandwidth, PED for FLAIR, and diffusion images. We observe issues in repetition time, pixel bandwidth, and magnetic field strength for TCGA-LGG and TCGA-GBM datasets. Missing acquisition parameters can not only limit reproducibility, it also limits the ability to perform comparative analysis across studies, which consequently affects the validity and reliability of the research. The compliance reports for TCIA are available at https://github.com/Open-Minds-Lab/mrQA-reports

**Table 3:** (a) An example reference protocol, and (b,c) compliance report for a toy neuroimaging dataset. Note that the reference protocol can either be pre-defined or inferred by searching for most frequent values for each parameter. The toy dataset has 7 modalities across 5 subjects. Subjects sub-566 and sub-879 are non-compliant w.r.t Pixel Bandwidth and Phase Encoding Direction respectively. For gradient-echo (GRE) modality, subject *sub-566* has a non-compliant pixel bandwidth for sessions 6-10

(a) Example of a reference protocol.								
Scanning Sequence	Echo Train Length	Phase Encoding Direction	Magnetic Field Strength	Phase Encoding Direction	Multi Slice Mode	Pixel Bandwidth	Flip Angle	MR Acquisition Type
GR	1	j	3	ROW	interleaved	350	40	2D
is3D	Phase Encoding Steps	Shim	Repetition Time	iPAT	Manufacturer	Sequence Variant	Body Part Examined	Echo Time
False	448	Standard	465	Grappa	SIEMENS	MTC_SS	BRAIN	3.73

(b) Summary of non-compliance for a toy dataset.								
Modality	Non-compliant		Non-compliant subjects	Reasons	Compliant		Total subjects	
rsfMRI	0	0.0 %			5	100.0 %	5	
GRE	1	20.0 %	sub-566	Pixel Bandwidth	4	80.0 %	5	
T2-FLAIR	0	0.0 %			5	100.0 %	5	
DTI	1	20.0 %	sub-879	Phase Encoding Direction	4	80.0 %	5	
T1-weighted	0	0.0 %			5	100.0 %	5	
SWI	0	0.0 %			5	100.0 %	5	

(c) Detailed report for non-compliance in GRE modality.								
Parameter	Ref. Value	Found	Subject_Session					
PixelBandwidth	350	485	sub-566_6, sub-566_7, sub-566_8, sub-566_9, sub-566_10					

## Appendix D: Effect of non-compliance in acquisition parameters

In this section, we discuss the impact on image quality due to variation in specific acquisition parameters.

### Flip Angle

The flip angle affects the net magnetization relative to the primary magnetic field as it controls the amount of longitudinal magnetization converted to transverse magnetization. Thus, the flip angle affects the RF signal of the cycle, thereby affecting the signal intensity. For instance, large flip angles produce T1 contrast, low flip angles produce PD (proton-density) contrast, and the T1 and PD contrast can cancel each other for intermediate flip angles (Sandmann et al., 2016; Balezeau et al., 2011; Lutterbey et al., 2007). Apart from anatomical MRI, the flip angle also affects functional MRI. Large flip angles deteriorate the spatial contrast between cerebrospinal fluid (CSF), gray matter (GM), and white matter (WM), which makes it difficult to align EPI to its structural counterpart (Gonzalez-Castillo et al., 2011). The impact on signal intensity and its significance for pattern discrimination would be governed by specific tissue in context. It is crucial to recognize that flip angles directly influence image contrast.

### Echo Time & Repetition Time

A correct choice for echo time (TE) and repetition time (TR) is important for structural images. The tissue-specific response can be influenced by acquisition parameters such as TR and TE as different tissues have varying T1 and T2 times. For instance, to generate a valid T1-weighted image, it is important that TR & TE is less than tissue-specific T1 & T2 times, respectively. In contrast, TR & TE should be much greater than tissue-specific T1 time & T2 time, respectively, to generate T2-weighted images. However, if TR is much greater than tissue-specific T1 time but TE is less than tissue-specific T2 time, the result is a proton density-weighted image. Thus, variations in TR and TE can dictate image contrast characteristics (Gold et al., 2004).

Apart from structural images, choice of TR and TE also affects fMRI images. Longer TE values lead to increased susceptibility artifacts and signal dropout. Shorter TRs provide enhanced BOLD senstivity but may also lead to saturation effects (Chen & Glover, 2015; Feinberg & Setsompop, 2013; Poldrack et al., 2008).

### Magnetic Field Strength

Variation in magnetic field strength has a strong influence on texture features (*e.g.*, co-occurence matrix and gray-level run length matrix). These texture features capture patterns and provide valuable information about visual appearance and structural characteristics of an image. These texture features can significantly impact the performance of predictive models.

Ammari et al. (2021) show a comprehensive analysis studying the impact of magnetic field strength on various texture features. The study evaluates 38 texture features of which 15 features in healthy volunteers were sensitive to variations in magnetic field strength. Visually, images from various field strengths may have the same visual diagnostic accuracy (Rutt & Lee, 1996), but in context to texture features, images with varying magnetic field strength cannot be used interchangeably (Ammari et al., 2021).

### Phase Encoding Direction

PED plays a crucial role in EPI sequences. A common issue in EPI sequences is their vulnerability to susceptibility artifacts. These artifacts are apparent, especially when the bandwidth is low (Jones & Cercignani, 2010; Le Bihan et al., 2006). To diminish the effect of these distortions, the collection of additional scans with varying PED is very helpful (Irfanoglu et al., 2012). Although it is important to eliminate these artifacts to improve image quality in DTI sequences, varying the PED is known to affect fractional anisotropy estimates.  Kennis et al. (2016) show that magnitude of fractional anisotropy magnitude between P$\gg$A and A$\gg$P scans can range from 0.4% to 30% even after correction for subject motion, eddy currents effects, and susceptibility artifacts. It is possible that these differences are arising due to signal intensities from P$\gg$A and A$\gg$P scans that can confound DTI neuroanatomical studies. Similarly,  Tudela et al. (2017) show that misalignment in PED from the main magnetic field can lead to much more artifacts reflected by lower fractional anisotropy values.

### Pixel Bandwidth

Increasing the pixel bandwidth provides the opportunity for shorter sampling time by allowing shorter TR as well as TE, that is especially useful for patients prone to head motion. Use of higher bandwidth in acquisition also reduces chemical shift and distortion artifacts (Graessner, 2013). However, increasing the pixel bandwidth not only leads to a significant reduction in SNR for the acquired image but also requires a higher field of view (Scheffler et al., 2018).

It should also be taken into consideration that definition of pixel bandwidth varies across scanner vendors. For instance, GE uses the bandwidth of the entire matrix (often measured in kHz), while the Philips scanners use the water-fat shift in pixels.

## Code Availability

The software package is available on the Python Package Manager (PyPI) at https://pypi.org/project/mrQA and its source code is publicly available at https://github.com/Open-Minds-Lab/MRdataset and https://github.com/Open-Minds-Lab/mrQA.
